# Developing HME-Based Drug Products Using Emerging Science: a Fast-Track Roadmap from Concept to Clinical Batch

**DOI:** 10.1208/s12249-020-01713-0

**Published:** 2020-06-22

**Authors:** Josip Matić, Amrit Paudel, Hannes Bauer, Raymar Andreina Lara Garcia, Kinga Biedrzycka, Johannes G. Khinast

**Affiliations:** 1 Research Center Pharmaceutical Engineering GmbH, Graz, Austria; 2 Institute for Process and Particle Engineering, Graz University of Technology, Graz, Austria; 3 Applied Manufacturing Science Sp. z o.o, Złotniki, Poland

**Keywords:** rational formulation development, hot melt extrusion, amorphous solid dispersions, process and product modeling

## Abstract

This paper presents a rational workflow for developing enabling formulations, such as amorphous solid dispersions, via hot-melt extrusion in less than a year. First, our approach to an integrated product and process development framework is described, including state-of-the-art theoretical concepts, modeling, and experimental characterization described in the literature and developed by us. Next, lab-scale extruder setups are designed (processing conditions and screw design) based on a rational, model-based framework that takes into account the thermal load required, the mixing capabilities, and the thermo-mechanical degradation. The predicted optimal process setup can be validated quickly in the pilot plant. Lastly, a transfer of the process to any GMP-certified manufacturing site can be performed *in silico* for any extruder based on our validated computational framework. In summary, the proposed workflow massively reduces the risk in product and process development and shortens the drug-to-market time for enabling formulations.

## INTRODUCTION

Active pharmaceutical ingredients (APIs) are becoming more potent and selective, resulting in increasingly complex formulations, and drug delivery strategies that are precisely tailored to achieve the required PK profile of a drug. Typical examples include poorly soluble APIs that require solubility enhancement (1–3). Moreover, advanced formulation strategies lead to more complex manufacturing processes, which increases the risk of development failure. In general, bringing a new drug to the market involves multiple time-consuming stages, with a go or no-go decision made at each stage. Since the pressure to bring a new drug to the market is immense, originators shy away from risky formulation designs and prefer simple drug delivery systems (DDSs), such as immediate release tablets. In order to counter this trend, our past work focused on de-risking the development and manufacturing stage of new and advanced DDSs**.** Examples include the development of small-scale formulation screening tools, *i.e.*, the vacuum compression molding (VCM) tool (4), advanced hot melt extrusion (HME) process models, mechanistic studies of biopharmaceutics, and stability aspects of enabling formulations and more, as described in detail in the sections to follow. Hence, we created a toolbox for rapidly developing hot-melt extruded formulations in tandem with the associated manufacturing process.

One approach to designing advanced formulations is solubility enhancement via amorphous solid dispersions (ASDs) made via pharmaceutical hot-melt extrusion (HME). HME is a potent production method, which is mostly used for the manufacturing of amorphous solid solutions and dispersions, as well as for dispersing and controlling the particle size distribution (PSD) of (nano-)crystalline APIs in polymer matrices (5–9). The resulting DDS can deliver both immediate and controlled releases (10–12), with or without biodegradable polymer matrices. Twin screw extruders (TSE) are most commonly used in HME, allowing flexibility during the process development. The process can be tailored by adapting the screw configuration and process parameters to match the critical quality attributes (CQA) of the drug. Several drugs produced via HME have been marketed to date, including Norvir® and Kaletra® (Abbott Laboratories), Onmel® (Merz), Noxafil® (Merck), Palladone® (Purdue Pharma), Viekirax®, Venclyxto® and Mavyret® (Abbvie), Eucreas® (Novartis), Zithromax® (Pfizer), Nucynta® (Janssen) and Nurofen Meltlets lemon® (Reckitt Benckiser Helathcare) and several implants and inserts, such as Zoladex® (AstraZeneca), Lacrisert® (Valeant Pharmaceuticals, USA), Depot-Profact® (Sanofi Aventis), Ozudrex® (Allergan, Ireland), and Implanon® (Merck, USA). The polymers typically used include HPMC, PEG, EVA, Soluplus, PVP, and Copovidone of various grades.

Besides HME, spray drying (SD) can be applied for manufacturing enabling formulations, *e.g.*, amorphous solid dispersions (13). In both HME and SD similar approaches are employed for formulation/excipient selection in terms of biopharmaceutics and stability performance of polymeric ASD. However, the processability requirements for the selected formulation candidates vary vastly since in SD solvents are added, which can alter and control the physical structure of the product. Focusing on overall aspects of HME-based formulation development, this review includes SD when early screening of formulations is performed in order to obtain information about the processability of the formulations.

Despite the advantages of HME (*i.e.*, formulation processing without solvents, a small footprint of the system, an intensified nature of process, a low energy consumption, a continuous nature, and manufacturing complex products with predefined release profiles in a single step) the vast majority of drugs on the market is made using other technologies. Moreover, several downstream options exist that enable companies to make tablets (calendering), powders (strand milling), and pellets for capsule filling of both spherical and cylindrical pellets (hot-die-face cutting or strand cutting).

There are several reasons why the adoption of HME is not much wider. First, HME does not have a long-standing history in the pharmaceutical industry, and, as such, there is a lack of experienced formulators and process engineers. Second, the development of HME-based formulation is considered risky and requires a significant expertise. Since despite the added benefit to the formulations (*e.g.*, solubility enhancement and defined release profile) HME may be too risky for the originators, such traditional approaches as micronization and functional coating are preferred. Third, the design of screws and the necessary scale-up is still performed mainly empirically for lack of sound design and scale-up framework. Lastly, the process flexibility poses significant challenges when dealing with new formulations and scale-up since the process window is not known *a priori* and has to be defined for every new formulation and extruder. Under the traditional approach, the formulation development is more or less detached from the process, *i.e.*, the biopharmaceutical requirements are met from the formulation standpoint while the processability, and the influence of process scale on the final product are not known. As a result, lengthy product development process is common, with multiple failed attempts leading to an unfavorable risk profile. Hence, integrated research, which takes into account formulation development from the pharmacological and processability standpoint, is needed for a “right-first-time” drug-to-market path.

**Fig. 1:**
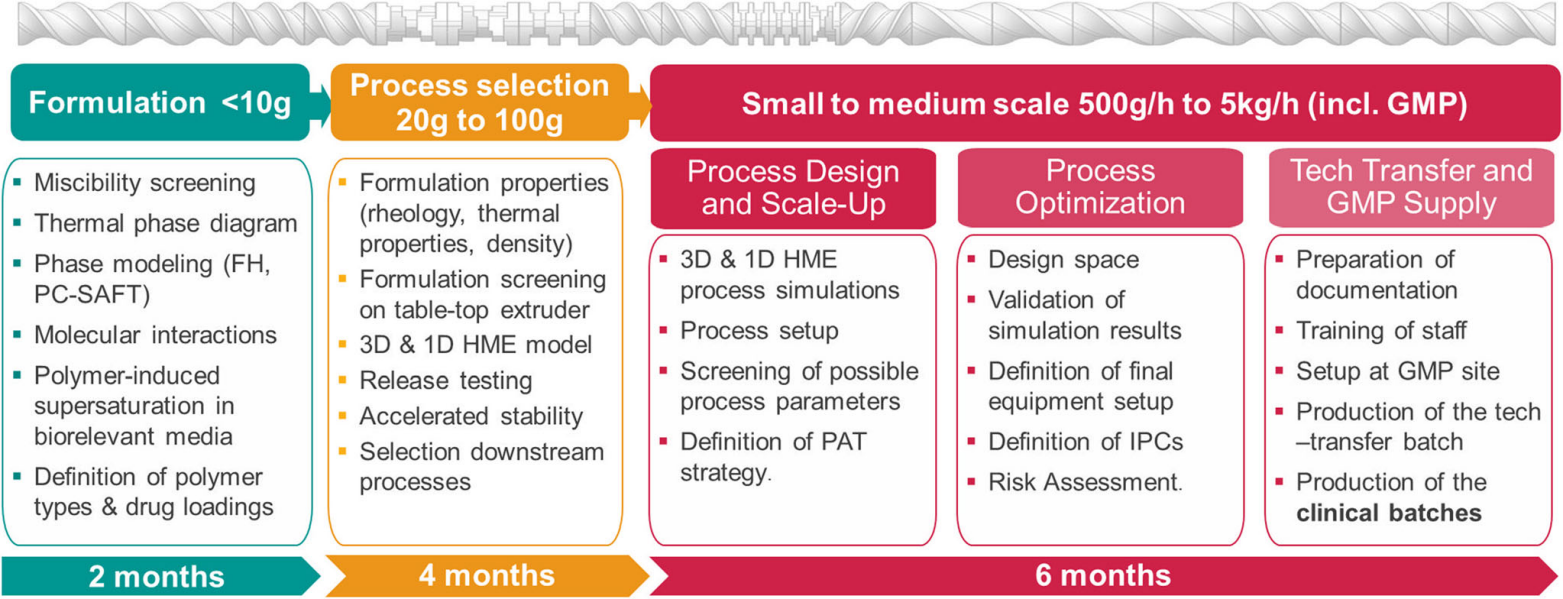
Integrated HME product development scheme

To that end, for many years our group and some others have focused on developing scientific tools that allow a fast and minimum-risk development of HME-based formulations using several advanced tools. A good recent review of these efforts is provided in (14). These include (1) advanced material science and screening, (2) small-scale test beds for formulations, (3) the design of small-scale processes, and (4) the scale-up to GMP production of clinical batches. Since most of the scale-up are performed empirically, one of the goals of our group was to create *in silico* tools for a rational, science-based scale-up, while addressing other important aspects, such as an API degradation. Our multi-step approach is shown in Fig. 1 together with the amount of materials required and the corresponding timelines.

All this is embedded in a quality-by-design (QbD) framework, including the definitions of quality target product profile (QTPP) and critical quality attributes (CQAs) of the drug product, a rational risk-based product and process development process, stability assessment and stability prediction, design space determination based on *in silico* and experimental tools, a control strategy based on risk assessment that includes specifications for the drug substance(s), excipient(s) and drug product and process capability and continual improvement (15–18). Ultimately, clinical batches are manufactured according to GMP.

As Fig. 1 illustrates, the formulation development requires a few weeks using less than 10 g of API. The process selection, including stability assessment and biopharmaceutics, takes a few months and less than 100 g of API. Finally, the process development can be performed rapidly using our advanced process design and scale-up framework. After about 6 months, the first clinical batch can be released. Details of the development process are provided below.

## PRODUCT DEVELOPMENT GUIDED BY QUALITY BY DESIGN PRINCIPLES

According to the ICH, quality by design (QbD) is a systematic approach to the development of pharmaceuticals that is based on sound science and quality risk management, with an emphasis on predefined objectives, product and process understanding, and process control (15,16,19–24). In the language of QbD, predefined objectives are reflected in the definition of the quality target product profile (QTPP) with the goal of achieving the intended therapeutic outcome and in the identification of critical quality attributes (CQAs). The importance of this first step cannot be overstated since all of the following product development efforts aim to satisfy the predefined route of administration, delivery system, dosage form and strength, targeted *in vivo* drug release, and pharmacokinetic profile as part of the QTPP requirements. Moreover, to ensure the desired product quality measured *via* the CQAs physical, chemical, biological, and microbiological properties should be within the appropriate limits. Preformulation studies, formulation design, and *in vitro* characterization focus on matching the final product’s QTPP. However, various process-related technological parameters of API and excipients need to be specifically considered as well.

**Fig. 2:**
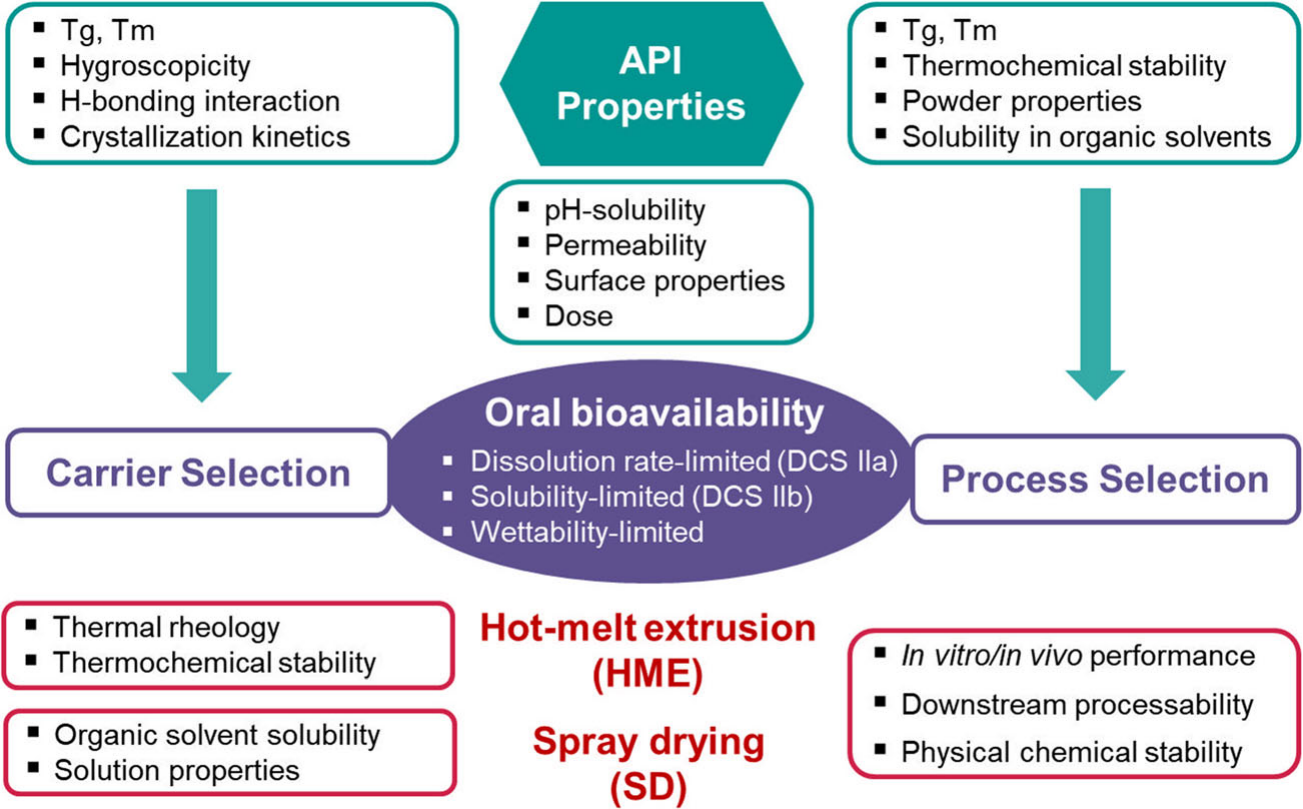
Basic physicochemical requirements for developing polymeric amorphous solid dispersions (ASDs) of poorly soluble drugs *via* HME and/or SD

Figure 2 provides an overview of important parameters for developing bioavailability-enhancing formulations of a poorly-soluble drug molecule *via* HME and SD. For example, pH-solubility profile and intestinal membrane permeability of a drug molecule define the class of the drug in biopharmaceutics classification system (BCS) (25). The molecules belonging to BCS class 2 and class 4 are poorly soluble, and their gastro-intestinal (GI) absorption can require solubilizing formulation concepts, such as ASD, lipid-based, or nano-crystals formulations (26). The BCS parameters need to be normalized by the intended dose of the given molecule, leading to the developability classification system (DCS) (27). The absorption of orally administered DCS IIa drugs is limited by the dissolution rate and that of DCS IIb is limited by the solubility. In some cases, poor solubility originates from the surface wettability of drug crystals.

Besides biopharmaceutics properties, the basic physicochemical properties for designing ASD of a drug molecule are the glass transition temperature, glass formation propensity, hydrogen bond donor/acceptor in the structure, melting temperature, and thermo-chemical stability. With regard to the ASD carrier selection, it is equally important to consider the properties of excipients. In the context of HME as a prospective technology, the drug and the polymers must have inherent thermal stability within the expected processing temperature. Since most pharmaceutical polymeric excipients are chemically stable at up to 200°C, high melting point drugs require either a higher intrinsic solubility in the selected polymer, or adding plasticizers to enhance their solubility in the polymer. Thermal rheology of polymers or selected formulations is decisive for processability *via* HME. For example, higher intrinsic viscosity and glass transition temperature of such cellulosic polymers as HPMC necessitate the use of plasticizer for extrusion.

The next step under the QbD approach is achieving a scientific understanding of the interplay between the product quality (CQA) and the process characteristics, *i.e.*, identifying the critical material attributes (CMAs) and critical process parameters (CPPs) and, most importantly, establishing the functional relationships between the CPPs, CMAs, and CQAs, which may be a scientifically most demanding and most vulnerable part of the product development. Traditionally, the assessment of product quality relies on complying with the product’s release specification criteria rather than designing the product by performing an appropriate risk assessment and defining a proper control strategy (19,20,28,29). The reason is often insufficient process understanding, especially with regard to complex processes that are borrowed from other industries and require a different formulation and process development approach than more traditional routes, as in the case of HME.

For HME purposes, the CPP-CMA-CQA relationship is typically established *via* extensive experimentation (currently performed based on DoEs), with a change in the CQA evaluated in terms of a change in the process settings, accompanied by elaborate statistical models that define the process windows. This approach, although widely applied, has a number of disadvantages, *e.g.*, poor predictability and impossibility of proper process transfer and scale-up since the process windows established are only valid for one formulation and one extruder under the exact conditions tested. Any departure from the formulation, equipment or process setting impairs the predictability and often requires a new set of experimental studies. This might be the single most important reason why HME is still not commonly used in the pharmaceutical industry.

The key to solving this problem is a proper definition of CPPs. In the case of HME, the list of process parameters currently considered to be critical is limited to the screw speed, the throughput, the barrel temperature, the screw configuration, and the die design. Although these process settings are good candidates for the CPP list, they affect the product quality only indirectly. Thus, establishing a control strategy for these settings alone cannot be a sufficient guarantee of the product’s quality. This is most evident during an HME process scale-up. The methodology traditionally has aimed to directly transfer the processes settings (mainly the screw speed and the throughput) from the original to the target scale using a geometrical factor that represents the change in the scale (typically the ratio between the outer screw diameters in some weighted form). However, this approach is not always successful. In the case of HME, the product CQAs, such as the degradation profile, are a result of the thermomechanical load cycle that the formulation experiences during the production. Hence, the proper CPP definition for HME must take into account the process states resulting from the process settings, *e.g.*, the axial distribution of average and peak melt temperatures, the overall and local RTDs, and the axial SMEC distribution (30). Only mechanistically based extruder models yield this kind of information. Machine-learning algorithms cannot be applied since they are based on data for one setting and formulation, which makes extrapolation and scale-up arbitrary.

It is important to note that in the event that proper CPP/CMA/CQA connections are established, it is comparably easy to go back to the product development if, for example, the required long-term stability of the amorphous form is not given. In this case, the manufacturing process or formulation can be adapted. Moreover, process control and quality risk management are significantly simplified as well.

An overview of HME-based product design is provided in the next Section, covering the formulation development, the process screening and the stability assessment. Process development and scale-up as well as the GMP production of clinical batches are covered in "PROCESS DEVELOPMENT AND CONTROL".

## FORMULATION DEVELOPMENT

**Fig. 3:**
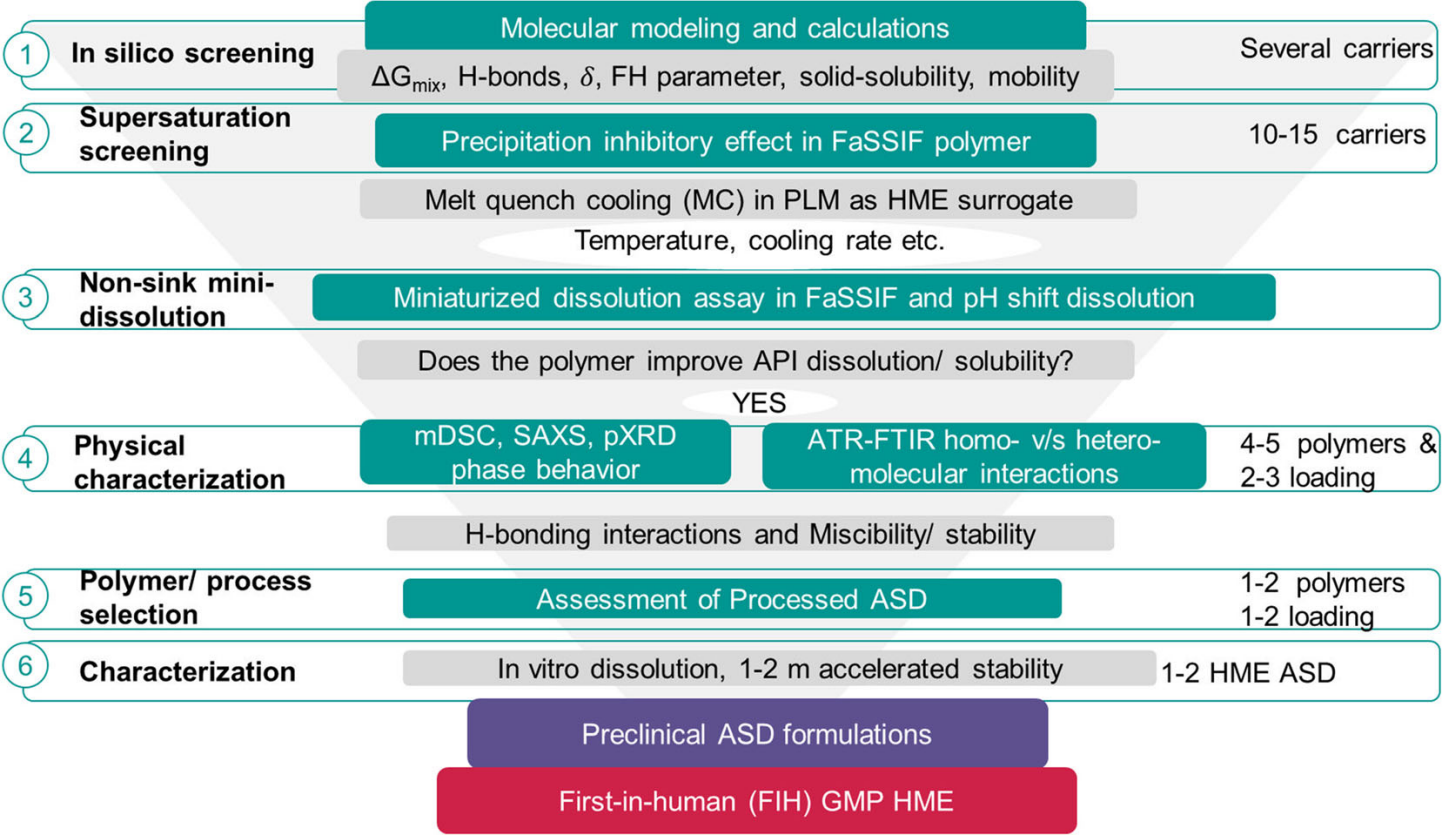
A systematic approach to potential carrier (polymer, surfactant, and combinations) selection for HME-based amorphous solid dispersions

Our approach to developing an enabling ASD formulation for a poorly soluble drug candidate *via* HME consists of (A) formulation and processability screening, (B) predictive computational and experimental methodologies for assessing biopharmaceutics and stability, and (C) advanced scale-up methods. This includes state-of-the art practices currently applied in industries in combination with emerging knowledge from academia. It should be emphasized that most of the workflow is equally applicable to the ASD development for manufacturing routes other than HME, such as spray drying (SD), milling, congealing, or supercritical fluid technology (Fig. 3).

## INTEGRATED PRODUCT DEVELOPMENT AND PROCESS SCREENING

Early phase product development is expected to balance the biopharmaceutics and stability targets and the manufacturability requirement for a given drug molecule. More precisely, the formulation candidates that are transferable from preclinical *in vivo* studies to first-in-human (FIH) dosing require systematic and thorough preformulation studies, screening and small-scale prototype preparation, which take into account the limited availability of drug candidate and the stringent development timeline. The preformulation screening is intended to provide the relevant information on biopharmaceutics, stability, and processability as early as possible.

We applied an integrated product and process screening framework that connects the formulation design (*e.g.*, carrier selection, drug loading) and the process screening (*e.g.*, HME, spray drying). Figure 3 shows the flowchart with a systematic 6-steps approach, combining theoretical calculations with the experimental screening of preformulation. First, a thorough theoretical calculation is performed using the molecular and intrinsic structural properties of the drug molecule selected. The goal at this stage is to set up an *in silico* formulation screening such that the experimental screening in terms of carrier types, their combinations, and drug loading can be rationally narrowed down in order to minimize lengthy experimental evaluations. The excipients included in these theoretical calculations comprise diverse ASD polymers and surfactants and their combinations. At this stage, molecular miscibility between the selected drug and polymer pairs or in the ternary system, including surfactant/plasticizer, is estimated using the solubility parameters of individual components. The total or partial components (dispersive, polar, and H-bonding) of Hansen’s solubility parameters (δ) of the selected drugs and carriers are estimated *via* group contribution methods. With the values of δ for different functional groups available in classical polymer chemistry text books, these calculations can be simply made manually using Excel spreadsheet. Alternatively, commercial tools such as MMP (www.norgwyn.com/mmpplus.html) can be used for this purpose. These values are further applied to assess the extent of drug-polymer miscibility. To that end, a simple and traditional approach, such as Greenhalgh classification, is employed with the purpose of obtaining qualitative values of miscibility (31). For the miscible pairs and ternary systems, Flory-Huggins (FH) interaction parameters are calculated using solubility parameters of individual components. For ternary systems containing a drug and a polymer and a second polymer or surfactant, ternary miscibility can be obtained *via* the vector distance among mixing components in the Bagley plots of partial solubility parameters (32,33). If the glass transition temperature of the selected drug molecule is already available at this stage, a theoretical composition-*versus*-glass-transition temperature profile is created based on ideal mixing theories, such as the Gordon-Taylor approach. The outcome of this stage will guide the selection of carrier combinations for the next stage. These theoretical inputs are periodically updated as the work progresses further. For example, other descriptors (*e.g.*, mixing energy, molecular mobility) are estimated *in silico* for promising systems using more advanced calculations, (*e.g.*, molecular dynamics simulations).

In step 2, high-to-medium throughput screening is performed to evaluate the excipients’ solubilization and supersaturation potential for a poorly soluble drug molecule with given physicochemical properties. The excipients include a range of polymeric carriers (PVP series, HPMC series, methacrylate series, *etc.*) and surfactants/plasticizers (SLS, tween, polysorbate, *etc.*) that are commonly used for ASDs and are broadly/qualitatively found to be miscible *in silico* from step one. Based on the experimental and/or predicted equilibrium solubility of the drug in the simulated physiological media selected (such as fasted state intestinal fluid, FaSSIF), a certain degree of supersaturation of the dissolved drug is induced in the medium containing pre-dissolved polymer of a given concentration. Supersaturation can be created via solvent shift (*e.g.*, introducing the drug solution into DMSO into FaSSIF), pH shift, temperature shift, *etc.* (34). The depletion kinetics of supersaturation in the biorelevant medium is monitored using the time-dependent turbidity measurements. The dissolved concertation is analyzed via chromatography. The data generated enable rank-ordering of excipients based on their supersaturation maintenance capacity for a given drug molecule.

Once a set of biopharmaceutically promising excipients is selected, in step 3, the miniaturized formulations are prepared *via* melt quench cooling to represent HME formulations. Alternatively, solution casting can be used to represent spray dried formulations (35). The cast film formulation can be prepared at a milligram scale for each drug loading using either glass well plates at a high/medium throughput temperature-controlled stages or DSC pans. Given sufficient time and resources, such screening can be performed in more process-mimicking setups: for example, levitated single droplet drying, oven evaporation at varying temperatures, or spin coating can be used to mimic spray drying (13). In addition, for HME formulation screening, vacuum compression molding (VCM) (4), thermal rheometers (36), or heated glass syringes with bent needles (37) can be employed to prepare mini-formulation samples to account for the extent of shear forces during HME. Varying drug loads are used until trace crystallinity is detected *via* polarized light microscopy. *In vitro* drug dissolution in these mini-formulations is assessed in a miniaturized way by directly introducing a biorelevant medium onto the surface of films and periodically sampling and analyzing the dissolved drug. This test can verify the results obtained from the supersaturation experiments and swiftly establish the effect of drug loading on the dissolution performance.

In step four, the mini-formulations containing a range of drug loads that resulted in a promising dissolution performance are further characterized in terms of their solid-state properties as follows: the glass transition and the degree of molecular mixing (one T_g_ versus multiple T_g_’s) via calorimetric analysis (DSC); the drug miscibility and the lack of crystallinity via X-ray amorphous halo (XRPD); the presence and strength of stabilizing molecular interactions between the drug and the excipient in the formulation (*e.g.*, hydrogen bonding, dipolar, and ionic interaction) via spectroscopy (infrared and/or Raman) and wettability *via* contact angle methods. In addition, the rheological measurements, the specific volume, the heat capacity, and the thermal conductivity are used to parametrize the models for the *in silico* assessment of the formulations’ processability, as discussed in more detail below. This systematically guides the selection of excipient and drug loading that maximize the dissolution performance and the drug-excipient miscibility to ensure the physical stability and processability in terms of thermal rheological profiles of the formulations selected.

In step five, based on the ranking of biopharmaceutics and the solid-state outcome, ASDs with the drug loading selected are prepared on the laboratory-scale HME using the carrier(s) selected. The information on thermal transitions (glass transition, heat capacity, melting, recrystallization, dehydration. *etc.*) obtained *via* DSC and thermal rheology allows to rapidly select the optimal process parameters (*e.g.*, the temperature profile of the extruder’s elements). Typically, a few dozen of grams of ASD powders are prepared at this stage. The ASD extrudates generated are milled using a laboratory scale ball mill or other impaction mills with a capacity to handle the lower batch size. Depending on the mechanical properties of the extrudate, either cryogenic or room temperature milling is performed. Some basic process parameters (*e.g.*, the milling intensity and time, the sieve size) can be varied to obtain ASDs of various particle sizes. Based on the information on the physical properties of the formulations selected, including the moisture sensitivity (which depends on the polymer/surfactant types), the processing operations may have to be performed under the reduced/controlled RH conditions.

The HME ASD formulations prepared in step five are thoroughly characterized in terms of solid-state, *in vitro* dissolution and short-term accelerated stability (typically 1–2 months) in step six. Depending on the intention and the development stage, milled HME ASD powders or powders compressed in tablets or filled into hard capsules are used. For example, if the dosing in the preclinical animal species is planned as an ASD suspension, the test also includes dissolution/supersaturation in the suspended state. The dissolution test at this stage includes the biorelevant media and transfer methods (pH/media shift from mimicking the gastric to intestinal environment), typically under the non-sink conditions. In addition to the milled powder and tablet/capsules of ASD, a physical characterization of the solid state is performed for the unmilled extrudate to ascertain the physical structure integrity during milling. The purpose of accelerated stability test at this stage is exploration rather than the prediction of actual shelf life. The propensity of crystallization/phase separation in the ASD candidates with promising *in vitro* performance is tested by storing them in an accelerated environment at elevated temperatures and RH, *e.g.*, 40 °C/75%RH. The test is performed under both open and closed conditions, with samples periodically withdrawn and tested via physical characterization and *in vitro* dissolution.

Based on the results of the small-scale formulation analysis, including the accelerated stability in step six, the formulations are selected for preclinical *in vivo* studies and/or FIH dosing for clinical programs. In (38), we provided a summary of a case study of a drug candidate screened for HME ASD using the aforementioned approach. In this case, for a poorly soluble new chemical entity (NCE), an *in silico* formulation screening for the carrier selection was performed based on the chemical structure. Following the results, supersaturation kinetics in FaSSIF were studied in about 16 combinations of polymer and surfactant carriers. The outcome of this study led to the selection of 4 formulation systems, *i.e.*, HPMC, Soluplus®, HPMC-AS, and HPMC-AS/HPC combinations. Subsequently, mini-formulation surrogates containing several drug loadings were prepared *via* solvent casting (SC) and melt casting (MC). The solid state characterization of these MC and SC formulations was performed focusing on miscibility and crystallinity; a non-sink dissolution study of intact films was performed as well. The ranking of performance based on the data led to the selection of two formulations, one with HPMC and a second one with Soluplus® and two drug loadings each. Finally, these formulations were produced as powders using laboratory scale HME (and SD) for the characterization of biorelevant *in vitro* dissolution and short-term accelerated stability. Based on the data, the system with HPMC with a given drug loading was selected as FIH formulation candidate with the Soluplus®-based system as backup. A similar step-by-step approach that is less rigorous in terms of bio-predictive and stability aspects was recently published by Simões et al. for etravirine HME ASD (39).

The adoption of such a systematic approach makes it possible to meet the development timelines using limited API amounts at the early stage. The entire screening stage can be accomplished within 4 months or less and using fewer than 100 g of API, depending on the complexity of physicochemical portfolio of the given drug. A thorough solid-state and biopharmaceutics characterization during the screening stage de-risks the development program. Rationally selected stabilizing carrier types and drug loading ranges that account for biopharmaceutics and processability provide a robust basis for interchanging drug loads (from low to high dosage strengths and vice versa), processing routes (HME to SD and vice versa), and iterating downstream processes and final dosage presentations (*e.g.*, powders or pellets filled in capsules versus tablets). The results of the screening phase provide the material properties and the formulation-specific data as an input for the model-based HME process development during the process setup, transfer, and scale-up. Moreover, the formulation properties data, such as the dissolution and stability performance determined using mini-formulations, can guide the parameter selection when developing predictive process models for the product performance. The industrial use of ASD preformulation and formulation development generates an enormous amount of data using identical approach for several NCEs. To this end, application of machine learning and artificial intelligence can further assist reducing future experimental efforts for the decision making (40).

## STABILITY ASSESSMENT AND PREDICTION

As one of the key quality attributes, stability of pharmaceutical products has to be ensured for the patient safety and efficacy. Being able to predict stability by combining the experiments and *in silico* modeling can drastically shorten the development timeline, while reducing the risk of re-formulation. Although empirical models based on Arrhenius kinetics are widely applied in practice for theoretical shelf-life prediction, they are limited to simple formulations and to cases in which instability can be readily conjectured based on the functional groups involved (*e.g.*, Milliard reaction between lactose and amine-containing drugs). In particular, with regard to ASDs the typical routes of instability are of both physical and chemical nature. On the one hand, amorphous phase separation and nucleation/crystal growth of active components of ASDs eliminate the expected solubility advantages. On the other hand, higher energetics and mobility of amorphous drug molecules in ASD prompt faster drug degradation and drug-excipient chemical interaction. Thus, an accurate prediction of stability in the final dosage forms is still challenging.

Ensuring physical stability of ASD requires a knowledge of both thermodynamic and kinetic factors governing (in)stability (41–47). From a thermodynamic standpoint, it is imperative to estimate the equilibrium solubility as accurately as possible, as well as, the kinetic miscibility of a drug molecule in a given polymeric carrier as a function of temperature. Experimentally, solid solubility of a drug molecule in the polymer is obtained *via* thermal methods (*e.g.*, melting point depression in DSC, T_g_ versus composition diagram, moisture sorption experiments in a dynamic vapor sorption system (DVS)) and is based on the solubility in low molecular weight liquid analogues of the polymers (48–53). As the fluid-state properties of polymers acting as the API solvent are challenging to establish, experimentally determined solid-solubility are often severely over- or underestimated. Therefore, a high resolution characterization using solid-state NMR spectroscopy/relaxometry and/or X-ray diffuse scattering analysis is necessary to verify the accuracy of the estimated solubility/miscibility (54). Moreover, experimental drug-polymer miscibility studies can generally be complemented by theoretical modeling, *e.g.*, Flory-Huggins lattice theory and perturbed-chain statistical associating fluid theory (PC-SAFT) (44). Despite certain assumptions and limitations, these models can provide working thermal-phase diagrams of a given drug and polymer that are equally important for the processing temperature selection in HME. For example, the group of Sadowski has shown the applicability of PC-SAFT-derived thermal-phase diagrams to determining the drug-polymer solubility curves and miscibility gaps, even in the presence of moisture, and verified it using the experimental stability data for the ASDs containing physico-chemically diverse APIs and polymers (55–60).

A simpler, less accurate and faster approach to estimating miscibility is *via* the total or fractional solubility parameters of the mixing components based on the “like dissolves like” concept. Since the solubility parameter is the square root of the cohesion energy density, the proximity of these values for a given drug-polymer pair indicates miscibility. The solubility parameters can be decomposed into the partial parameters to represent dispersive, polar, and hydrogen bonding contributions. These solubility parameter values can be estimated using group-contribution methods or, if the molecular structure is known, a molecular dynamics (MD) simulation. Besides thermodynamics, various modes of molecular mobility (global and local motions) can contribute as a kinetic factor for triggering the phase separation and the drug crystallization. Global molecular mobility can be estimated *via* structural relaxation experiments using DSC, NMR relaxometry, dielectric spectroscopy (DES), and dynamic mechanical analysis (DMA), while local mobility is determined *via* DES and DMA. Since the average time scale for global molecular motion can be empirically related to the onset of crystallization for the given systems as a function of temperature and humidity, it can be used to predict the physical stability (61).

Molecular dynamics (MD) simulations and first principle methods are increasingly applied to rationally develop a stability ranking based on both thermodynamic and kinetic factors (62). For example, we recently employed MD simulations to investigate the relative contribution of thermodynamic factors (Gibbs free energy of mixing and hydrogen bonding interactions) and kinetic factors (diffusion coefficient and roto-vibrational mobility) to the physical stability of ASDs (63). Comparing the outcome of MD simulations to the experimental stability data made it possible to define the prominent effect of molecular mobility on the stability in systems with a lower intrinsic molecular miscibility. Initially, the MD simulation-based approach appears to be slower and more costly. However, once the necessary force fields are created for common ASD polymers, they can be used repeatedly for ASDs of new drug molecules with a minimum effort required for obtaining a rational stability ranking. Such a prediction framework has been applied to various NCEs and ASD candidates undergoing clinical developments.

In terms of chemical stability of ASDs, the predictive methods are still limited to empirical extended Arrhenius kinetics or statistical approaches, mainly due to the complexity and insufficient understanding of the mechanisms involved. However, to accurately predict the ASD stability, models that combine both physical and chemical transformations are required. On the experimental side, drug-excipient compatibility studies for developing HME-based ASDs need special attention so that any process-induced incompatibility can be ruled out as early as possible. A typical concern with this regard is reactive impurities (*e.g.*, free radicals, oxidizing species, and aldehydes) as a consequence of thermal treatment of polymers during extrusion and the drug’s susceptibly to such reactive species during and/or after production of ASDs (64). To that end, we recently applied the controlled pressurized oxygen heat space and temperature setup (RapidOxy^Ⓡ^) as a tool for rapid assessment of the chemical interaction between famotidine and PEG of different molecular weights and at different drug loads (65). The temporal oxygen pressure drop was used to estimate the consumption of oxygen *via* polymer degradation. The formation of reactive radicals and formaldehyde was confirmed *via* ESR spectroscopy and IR spectroscopy, respectively. This method allows assessing the incompatibility within a day while other approaches may take months. Furthermore, we are working on developing a scientific insight with regard to the generation of reactive impurities, their solubilities, and diffusion rates in polymeric excipient matrices with the ultimate goal of creating a predictive model for reactive-impurity–mediated drug degradation in ASDs.

## BIOPHARMACEUTICS ASSESSMENT

Biopharmaceutics of pharmaceutical products contain the most important parameters for ascertaining the success of a given formulation and processing strategy, including the *in vivo* absorption of drug molecule and the systemic availability. The basis for establishing the biopharmaceutics of a drug product is the dissolution process (and possibly recrystallization due to supersaturation *via* ASD) in the GI milieu and permeation of the dissolved drug molecules through the GI membrane *via* active and/or passive transport. These parameters are tested *in vitro via* biorelevant dissolution testing and drug permeability through the artificial membrane or cell membrane. Biorelevant dissolution testing uses the gastric fluid simulated sequentially over time, followed by the simulated intestinal fluid. The *in vitro* results, together with *in vivo* pharmacokinetic data, are used to construct *in vitro–in vivo* correlations (IVIVC) or to develop a predictive mechanistic physiologically based pharmacokinetic (PBPK) model *in vivo*.

In the case of ASD formulation, predictive *in vitro* and *in silico* biopharmaceutics characterization can help to secure the *in vivo* success by considering the excipients’ solubilization factors, supersaturation generation, and maintenance potential, precipitation inhibitory capacity in the GI environment, and contribution to accelerating or decelerating the drug permeation rate (66). It is common practice to perform *in vitro* dissolution of ASD formulations under non-sink conditions, meaning that the total drug amount in a given medium volume is several times higher than the solubility of the crystalline counter-part (67). The exact *in vitro*/*ex vivo* simulation of *in vivo* situations is challenging since disintegration, drug solubilization, ASD surface plasticization, and supersaturation are connected events. However, the reasonable accuracy obtained by combining an advanced *in vitro* characterization with *in silico* models helps the formulators to choose and/or to modify the functional excipients, drug loading, and processing parameters while scaling up the HME process for the production of clinical supplies (68,69).

Recently, we performed a systematic biopharmaceutics characterization of generic tacrolimus modified release ASDs (Envarsus® prepared by MeltDose®) in the form of tablets and compared these to the original ASD pellets in capsule formulation (Advagraf®) (70). By employing the non-sink dynamic and the biorelevant *in vitro* dissolution in combination with the *in vitro* cell permeability as inputs for the compartment PK model, the *in silico* drug plasma concentration time profiles were generated using GastroPlus®. The *in silico* data obtained were compared to the *in vivo* clinical trial data to establish an IVIVC model, which enables a comparison of the two formulations with respect to the predicted *in vivo* population PK profiles.

There are several other aspects associated to the biopharmaceutics of ASDs that require a better scientific understanding. More precisely, the complex interplay between the formulation, the process and the performance of ASDs requires an integrated evaluation of the detailed solid-state and surface characterization and a thorough biopharmaceutics characterization of the products (71). This way, *in vivo* predictive models can be developed to shorten the expensive clinical phases and replace bridging *in vivo* PK studies when either the formulation or the process is modified (*e.g.*, different grades of polymer, HME *vs.* SD, or different production scales). Moreover, the *in vitro* assessment of food effects using appropriate biorelevant media can help to establish a virtual bioequivalence when developing a generic product (72). Provided that there is sufficient *in vitro* data on dissolution and precipitation, the *in vivo* drug release profile of ASD can be described, combining drug dissolution, and nucleation with crystal growth models, which are yet to be incorporated into commercial PBPK models (73). Clinical data of HME-based drug products, including ASDs, are still rarely reported in literature (74). Currently process and product modeling are linked *via in vitro* data, *i.e.*, the process modeling aims to cover the process behavior and aspires to predict the *in vitro* performance of the drug, whereas product modeling aims to link the *in vitro* data and predict the expected *in vivo* behavior. Increased accessibility of the *in vivo* data for HME-based products will enable improving, as well as validating, the end-to-end predictive solutions applied for the product development.

## PROCESS DEVELOPMENT AND CONTROL

### Process Setup and Scale-up *via* Advanced Modeling

As mentioned above, HME has a number of advantages over traditional batch technologies in terms of process flexibility, footprint size, solvents requirements (or the lack thereof), and the possibility of single-step production (75–82). One of its most important benefits is the continuous nature of process, allowing a seamless integration of upstream and downstream units into the drug production process. Yet challenges still remain, which are mainly due to the vast number of parameters and screw designs. For example, there are no readily available design tools for a process involving a novel drug (or even a generic drug for that matter) that do not require extensive experimental efforts of an experienced extrusion process scientist. This is a problem since experimental DoEs have high material and facility costs and an unfavorable risk profile. To address this issue, we developed a rational design framework for twin screw extrusion HME processes and the corresponding downstream processing using novel *in silico* approaches. Specifically, we focused on:

- Predicting performance of individual screw elements and their effect on the fluid flow and dispersive mixing *via* detailed 3D simulations (30,83–85)**;**
- Quantifying the effect of (complex) screw configurations and various process settings on the melt temperature, fill ratio, SMEC and RTD *via* advanced, fully parametrized 1D HME simulations (5,30,86,87); and
- Including material CMAs and product CQAs (*e.g.*, crystallinity and degradation) into the modeling, allowing the process response prediction for an accurate process setup and scale-up.

**Fig. 4:**
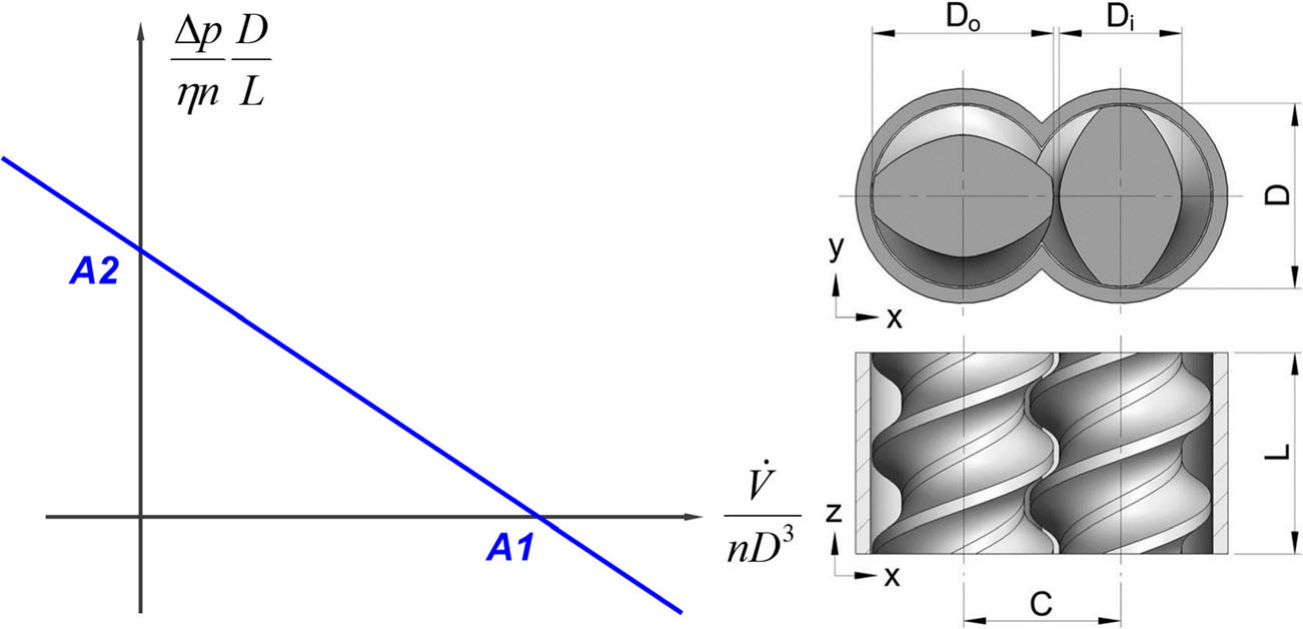
An exemplary plot of dimensionless pressure characteristic curve with A_1_ and A_2_ axis intercepts (left) and an example of a screw geometry for a conveying elements pair routinely used in the HME production (modified from (30))

Understanding the effect of screw geometry on the fluid flow, energy dissipation, and distributive mixing is crucial for the design on new elements and the assembly of screw configurations (75,76)**.** A typical cross section of a TSE screw pair is shown in Fig. 4-right. The cross section shows a pair of double-flighted conveying screw elements denoting their most important dimensions, like barrel diameter (D), outer (D_o_) and inner (D_i_) screw dimeter and the screw centerline distance (C). Our approach uses smoothed particle hydrodynamics (SPH), a relatively new numerical method for simulating complex free surface flows occurring inside the rotating screws. SPH is a Lagrangian-based fluid dynamics model, with the fluid flow represented as a continuum of moving fluid parcels that can adjust naturally to the complex intermeshing movement of twin screw extruders without a numerical mesh (88–95). The Lagrangian nature of the method also allows for a straightforward investigation of the flow in partially filled screw elements, as well as, a detailed investigation of the distributive mixing action of the screw geometry selected.

Using Newtonian fluids as a reference and assuming a creeping flow regime (low Reynolds number, *i.e.*, high fluid viscosity), the flow data can be analyzed in a simple dimensionless manner, describing the performance of any screw element pair regardless of the material, screw speed, and length of the screw element (30,76,83–85,96). Thus, the performance of any screw element pair can be described in terms of pressure and power characteristics. The pressure characteristics is a linear relationship (under the abovementioned assumptions) between the dimensionless volumetric throughput and the pressure build-up capacity that a certain screw pair possesses (76,83,85). Since the relationship is linear, the curve is sufficiently determined by the axial intersects termed inherent conveying A_1_ and the pressure build-up capacities, A_2_. The former represents the dimensionless volumetric throughput at zero backpressure, whereas, the latter is the theoretical dimensionless backpressure where no overall mass flow occurs. Pressure characteristics are provided in Fig. 4-left, with the *x* axis showing the dimensionless volumetric throughput and the *y* axis the dimensionless pressure build-up. The blue curve represents a typical pressure characteristics of a twin-screw extruder element pair, with its A_1_ and A_2_ axis intercepts. The non-Newtonian nature of fluids is accounted for separately as part of the 1D HME codes.

The power characteristics is a linear relationship between the dimensionless volumetric throughput and the power consumption, which can be described using axial intersects B_1_ and B_2_ (similarly to the pressure curve) (76,96). Over the past years, we have created a database of the most common twin-screw extruder elements of major pharma extruder manufacturers of various scales (12-mm, 16-mm, 18-mm, and 27-mm extruder sizes) (30,83–85). Since analyzing the flow and mixing behavior in such detail is computationally expensive, we developed an in-house software for accelerated simulations running on graphic cards (GPUs): the eXtended Particle Systems (XPS) software (97–109). It is a powerful simulation platform for simulating not only complex fluids *via* SPH, but also complex powders *via* the discrete element method (DEM) with additional coupling to conventional CFD software.

**Fig. 5:**
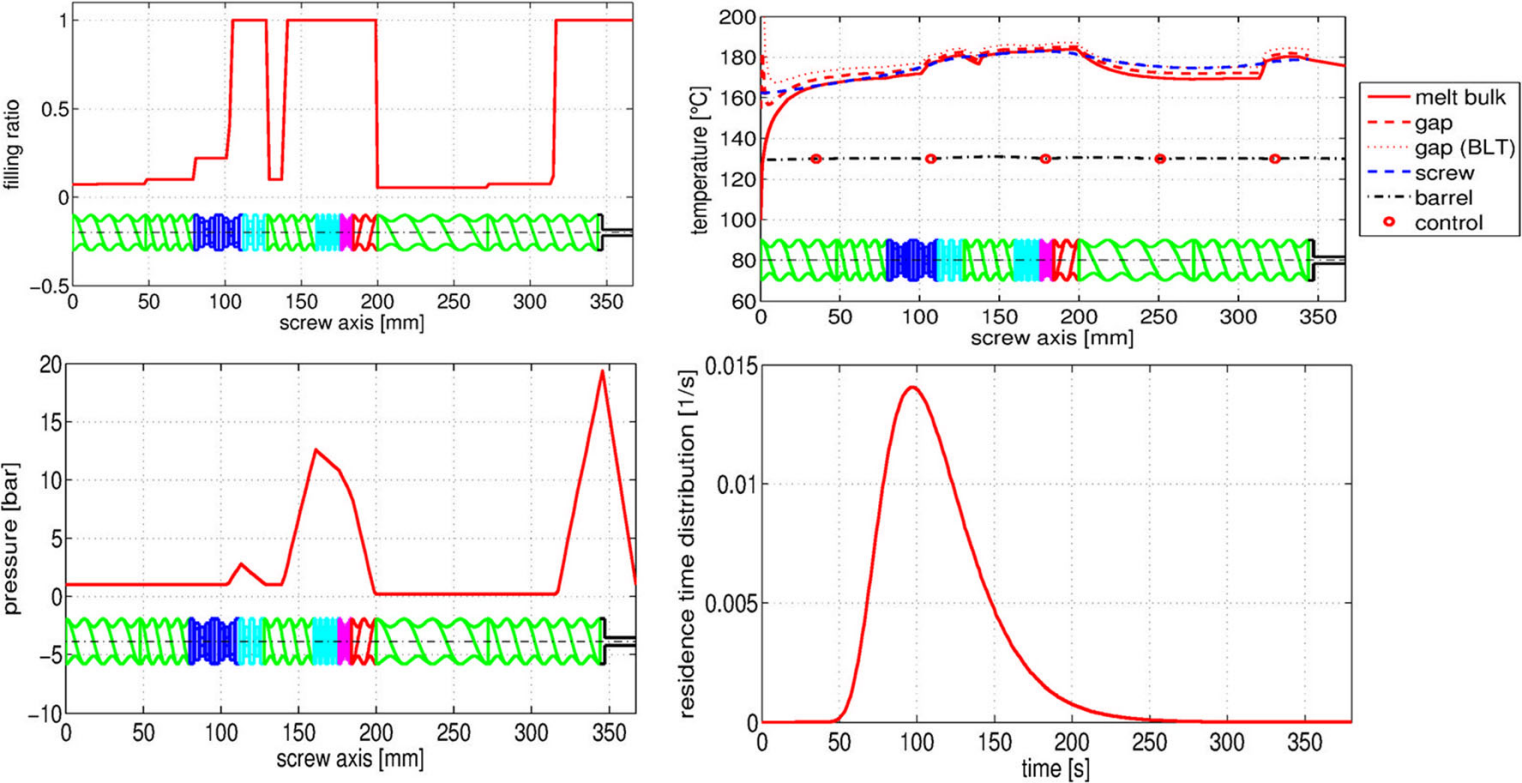
Example results of the reduced order 1D HME simulation software showing the axial melt-filling ratio (top left), axial melt temperature distribution (top right), the axial melt pressure distribution (bottom left) and the residence time distribution (bottom right) (110). The figures also show a color coded screw configuration. The green element are conveying elements, the blue elements kneading elements (dark blue being kneading elements with a 90° angle between the kneading discs), magenta represents mixing and red back-conveying elements

Although the speedup and cost effectiveness offered by a GPU platform are significant in comparison to conventional software, the complexity of the flow and material behavior makes SPH unsuitable for simulating the full extruder for industrial settings. Thus, to design a process for our industrial partners, we developed a reduced-order 1D HME model on the basis of lessons learned from the detailed SPH analysis (5,30,86,87). Some results of such a reduced order simulation are shown in Fig. 5, illustrating the fill ratio (top left), the pressure (bottom left), the melt temperature profiles (top right), and the residence time distribution (bottom right) at a certain rpm and throughput (starved feeding). This allows us to perform *in silico* DoEs using a variety of extruder setups, screw configurations, process settings, and formulations. From the process equipment standpoint, data acquired for the torque required for processing the formulation selected in the desired process settings make it possible to decide on the suitable extruder. Analyzing the thermal and mechanical loads (melt temperature, SMEC, local and overall RTD) to which the formulation is exposed to during the process can assist with the choice of formulation, process settings, and screw configuration for obtaining the desired product CQAs (16,30,75–77).

This allows us, first, to choose the adequate formulation candidates in terms of formulation processability. In the second step, the suitable equipment, screw configuration, and process settings can be selected. The product quality can be predicted even before transferring the process to the pilot plant scale. Our HME setup workflow consists of four steps:

1. Detailed analysis of the API and formulation candidates, including measurements of the formulation’s rheology, heat capacity, thermal conductivity, and specific volume, for the parametrization of 1D HME models.
2. A detailed analysis of the extruder’s screw elements *via* SPH, determining the power, pressure, and mixing characteristics of individual screw pairs, for a parametrization of 1D HME models.
3. An *in silico* DoE using the 1D HME model as a basis for determining the process response (torque, SMEC, melt temperature, RTD, *etc.*) as a function of the selected formulations, screw configuration variants, and process settings, with the goal of determining the most promising formulation candidates and process settings.
4. Validation and fine tuning of the process setup in the pilot plant and prediction of the product’s CQAs (including degradation and concentration).

**Fig. 6:**
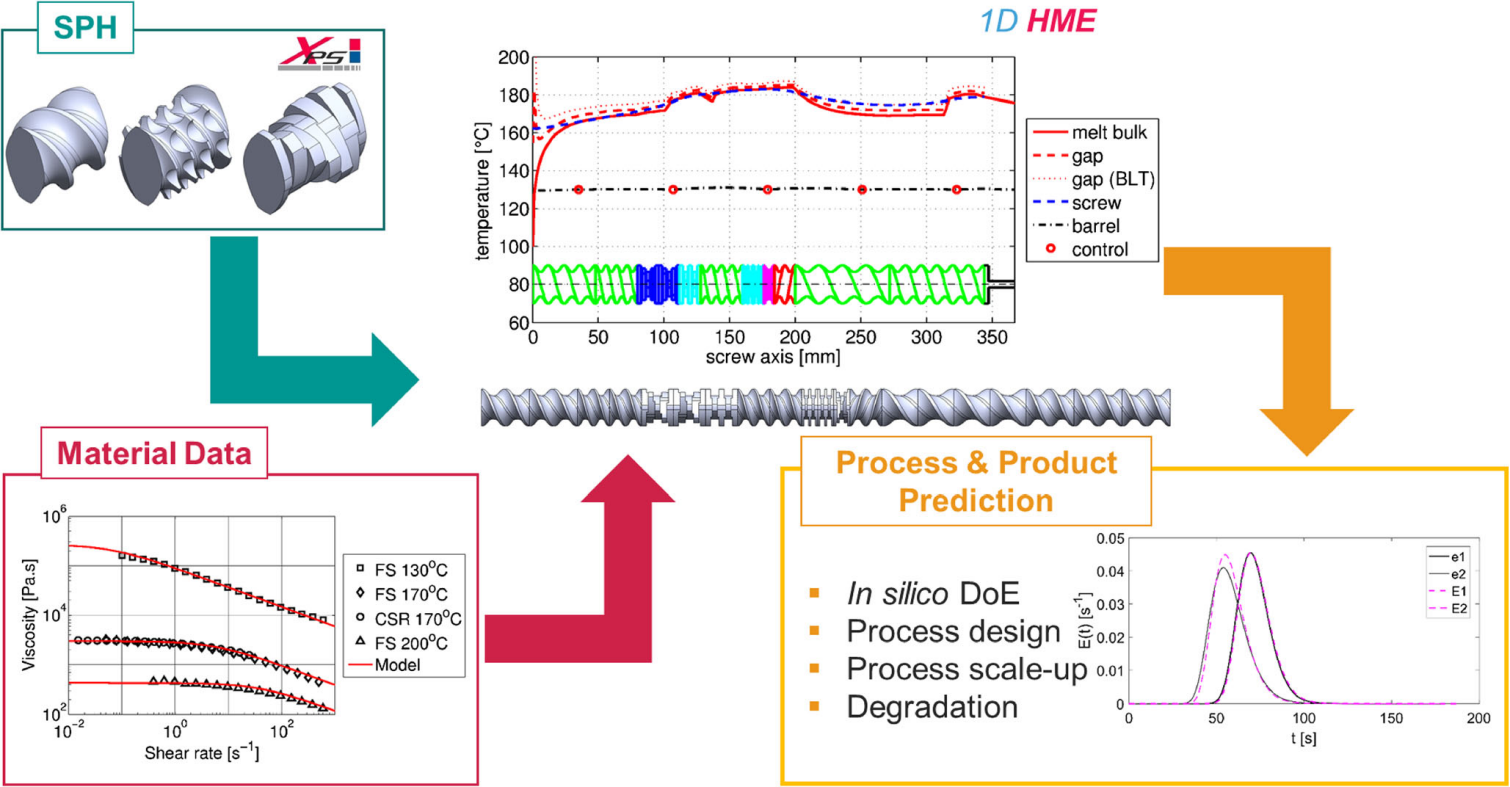
Steps for a quick and reliable HME process setup, including the investigation of material data (melt rheology, specific volume, heat capacity, and thermal conductivity), detailed 3D SPH simulations of individual screw-pair elements; 1D HME validated process simulations and process and product prediction

The computational approach is highlighted in Fig. 6. The characterization of the individual screw elements of the chosen extruder is done *via* the SPH simulation method (left top) (30,83–85). This includes the computation of the pressure and power characteristics, as well as the distributive mixing capabilities of the screw-element pair in a non-dimensional and formulation-independent manner, as discussed above. As such, the result reflects the geometrical capabilities of the screw element pairs and is in the next step used as descriptors in the 1D HME model (right top). In parallel to the SPH screw pair characterization, the API in question is analyzed and a suitable polymeric carrier is defined, according to the steps described in the formulation development section of this paper. Once the formulation is defined, the rheology, specific volume, heat capacity, and thermal conductivity of the mixture are determined. The data are then used in the 1D HME model (left bottom). Once the screw element pairs and the formulation are parametrized, a variety of screw configuration and process conditions can be tested and evaluated *in silico* (right top) (30,86,87). The obtained results range from axial distributions of the filling degree, melt temperature, pressure distribution, SMEC distribution to local and overall RTDs (local in the sense that RTD for only a certain screw section can be calculated, which is not possible to be done experimentally). In combination with experimental runs for the verification of the *in silico* results, it is possible to perform HME process setup and scale-up in an efficient and product-specific manner, taking into account the product CQAs (right bottom). Validation was performed on our fully-PAT equipped extruders on various scales.

An essential part of product development from the first formulation screening efforts to the clinical batch manufacturing is the HME process scale-up and transfer. Knowing all the relevant product-process relationships makes it possible to scale-up the process from one scale to another in a rational manner. The guiding assumption for every process scale-up and/or transfer is that the product quality is the result of defined thermomechanical loads (*i.e.*, SMEC, melt temperature distribution, and RTD) that the formulation experiences during the production. Thus, keeping the thermomechanical loads constant across the various scales and types equipment is crucial for a consistent product quality.

By using our novel scale-up approach based on 1D HME models, we are able to adjust the screw configurations and process settings to match the thermomechanical load profile on the original extruder scale (30). The great advantage of using *in silico* tools for process scale-up and transfer is that there are virtually no limitations in terms of screw configuration and process settings. Moreover, no material is wasted. Under traditional scale-up approaches, the prediction of process settings on the target scale is based on the process settings on the original scale multiplied by a geometrical factor representing the similarity between the scales. The thermomechanical load history is disregarded, which often necessitates significant additional experimental efforts with the goal of matching the product specifications. In addition, changing the extruder scale may require changing the extruder brand, which creates additional issues in terms of matching the screw configuration and the extruder capabilities. In contrast to the traditional approaches, 1D HME model directly calculates the axial SMEC and the melt temperature distribution together with the local and overall RTDs, accounting for the thermomechanical load history.

**Fig. 7:**
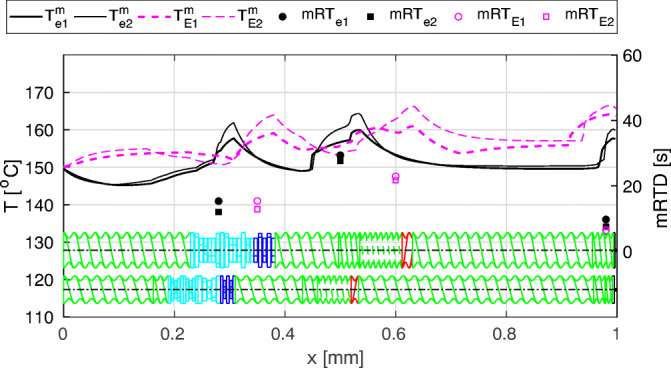
An example of axial melt temperature distributions and mean RTDs in various zones of screw configuration on two extruder scales. For more details refer to (30)

Figure 7 shows an example of axial melt temperature profiles (black is small scale, pink is large scale) and mean RTDs in the various extruder zones. The scale-up was performed to keep the peak melt temperatures in the kneading and mixing zones similar to the peak temperatures that the formulation experienced in the original extrusion setup. In addition, the goal was to assure that the mean RTD of the formulation in the (high temperature) kneading and mixing zones is equal or below the one in the original setup. This rules out any unexpected changes in the product quality (degradation). Hence, our approach directly aims to transfer the thermomechanical load history, regardless of the screw configuration, extruder scale, and extruder manufacturer. This way, full flexibility in terms of extruder manufacturers is attained, allowing to test various extruders *in silico* before purchasing the actual equipment.

Specifically, our approach includes:

- Precise assessment of extruder performance on different scales, from the formulation screening to pilot plant and production scales;
- Design and optimization of HME process together with a control concept;
- Rational scale-up procedures that are based on sound science, eliminating simplified rules;
- Prediction of in-process degradation profiles.

### Experimental Verification of Process Setup and Scale-up

Validation is a critical part of every model development. To that end, model results were validated *via* multiple experimental investigations across various scales. Table I lists the equipment used. Beginning with the formulation screening on the 9-mm ThreeTec table top extruder, moving to the pilot and clinical batch manufacturing scales using the 18-mm Coperion extruder and finishing with the full-production scale process development on the 27-mm Leistritz extruder, all the relevant pharma-scales were studied, and the models were validated. In addition, several downstream options available (see Table I) have been studied (110–113), including hot-die-face cutting, strand cutting, calendaring systems or mills, tableting or capsule filling equipment, allowing an initial manufacturing test of multiple dosage forms.

**Table I:** Extruders, Upstream, and Downstream Equipment Available at the RCPE Pilot Plant

Upstream equipment	
Ktron K20 feeders (0.5–6 kg/h)	
Brabender feeder (0.1–1 kg/h)	
RCPE’s micro feeder (1–100 g/h)	
HNP liquid pumps	
Twin-screw extruders	
Three Tec TT ZE9 9 mm—table-top extruder	
Coperion ZSK18 18 mm—pilot plant scale extruder	
Leistritz MIC27 27 mm—pilot plant & production scale extruder	
Thermo Fisher Pharma16 16 mm—GMP pilot plant extruder	
Single-screw extruder	
Brabender Compactextruder KE 19 19 mm–co-extrusion	
Downstream equipment	
Maag Ex 22–4 melt pump	
Maag Hot Die Face Pelletizer	
Automatik P60E strand granulator	
Gabler Engineering R-250 spheronizer	
Colvistec UV-VIS inline spectrometer	
Zumbach extrudate laser diameter measurement	

Moreover, embedding of a nano-suspension in a polymeric matrix (5,6) and co-extrusion using a twin- and single-screw extruder in combination can be modeled using this approach.

**Fig. 8:**
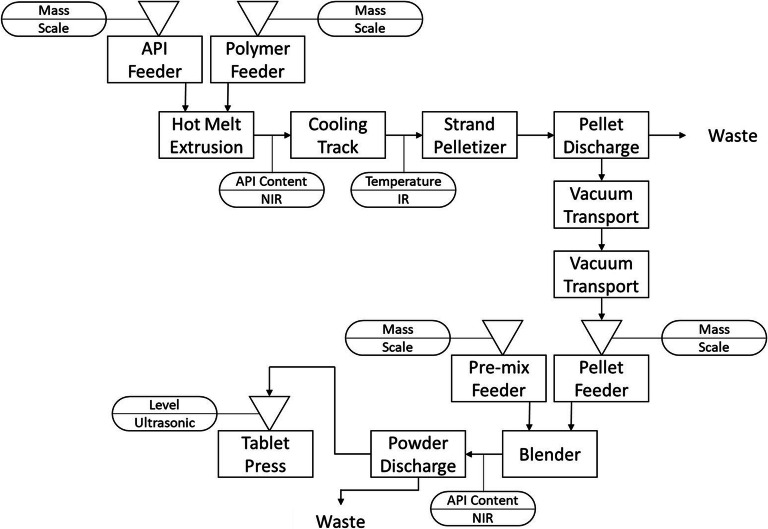
Process diagram and sensors for a continuous HME direct compaction manufacturing process (115)

Lastly, we established a continuous HME-tableting line, with HME used to produce an ASD and nano-based formulation. The strand is cooled and cut into small pellets that are fed to a continuous direct compaction line consisting of loss-in-weight feeders, a blender, and a tablet press (114,115). Moreover, a sophisticated model-based control concept was developed that allows the continuous manufacturing process to remain in a state of control while combining various production steps. Figure 8 shows the flow sheet of the process, the control systems, and the tools for dealing with the out-of-spec material.

### GMP production of clinical batches

After the formulation development, process setup and verification in a non-GMP environment, a transfer to a GMP facility can be made for clinical batch supply and product manufacturing. As mentioned above, properly establishing the CPP/CMA/CQA relationships greatly simplifies the subsequent process transfer to a GMP facility. Certain GMP activities, such as an evaluation and a qualification of source material, can actually begin before the manufacturing process development is completed. However, a deep understanding of CMAs and their link to CQAs should be achieved in order to control the source materials and especially the API.

Technology transfer is defined as a “logical procedure that controls the transfer of any process together with its documentation and professional expertise between development and manufacture or between manufacture site” (116). Ideally, the location of the clinical batch and commercial product manufacturing is selected before or in parallel with the process development. The reason for this is that the capabilities of the manufacturing facility (*e.g.*, type of equipment, screw configuration availability, batch size and/or throughput) have to be considered when developing the product for the reason mentioned above. If the capabilities of the receiving site differ significantly from those used during the development, timelines may be affected and a greater effort on re-development can be expected (21–23,116). Determining the setup and the control strategy based on the process state rather than the equipment type/setting makes scale-up and transfer activities less dependent on the receiving site’s particular equipment, hence, following risk management approach. This way, extensive experimental runs can be avoided, saving material, time, and use of GMP facilities. Even if the material specifications change during the transfer, a rational design model will be able to account for such a variability, eliminating trial, and error. In addition, a more mechanistic understanding of the process can be a great tool for assessing and supporting the product’s life cycle management.

Over the last years, RCPE has created a strategic partnership with AMS-Pharma in order to complement and support the development process up to clinical batch manufacturing under GMP. AMS-Pharma holds several GMP certifications for manufacturing operation and quality control activities of Human Investigational Medicinal Products and Human Medicinal Products. The facilities enable clinical batch manufacturing and commercial manufacturing supported by product and process development knowledge generated by RCPE’s scientific expertise. The HME clinical batch manufacturing is performed using a TS extruder (PharmaLab 16 TSE) and upstream and downstream processing equipment (Table II).

**Table II:** GMP Extruders Available at AMS-Pharma

Upstream Equipment	
Ktron K20 feeders (0.5 –6 kg/h)	
Twin-Screw Extruder	
PharmaLab 16 TSE Thermo Scientific	
Downstream equipment	
1.2 m conveyor belt, air-cooled	
Strand pelletizer, pellet length 1–3 mm	

## SUMMARY AND CONCLUSION

Drug product development is a complicated and risky endeavor, especially with regard to complex enabling formulations, such as ASDs made *via* hot-melt extrusion or spray drying. To facilitate it, we developed workflows and platforms for rapid product development that allow a rational design of formulations, process and scale-up/tech-transfer to GMP manufacturing within less than a year. The first step (Fig. 1) is screening the suitable carriers and establishing a detailed understanding of API-carrier interactions, which allow an analysis of long-term stability and biopharmaceutics of the products. Both theoretical tools (*e.g.*, MD simulation, PC-SAFT modeling, Flory Huggins model, Gordon-Taylor equation) and experimental screening methods (DSC, rheology, *etc.*) form a (semi-) predictive framework for a rational formulation development. Accelerated stability screening and detailed analysis of biopharmaceutical parameters (*e.g.*, biorelevant supersaturation and *in vitro* dynamic dissolution) are the next logical step. The outcome of these efforts is the selection of suitable carriers for a specific API formulation (for example ASDs). In the past, we demonstrated that such a rational formulation development workflow can be completed within a few months.

Once the formulation has been fixed, the carriers have been selected and the degradation profile has been established, the process development can be performed *in silico* based on detailed rheology data (Fig. 6). Lab and pilot plant extruders are used to verify the model-based selection of processing conditions and screw design. If the screw parameters are available, simulations can proceed swiftly. If not, they have to be established *via* detailed SPH simulations (for our extruders, the screw parameters were known). Altogether, this process can be completed within a few months.

The last step is the process transfer to the extruder at a GMP-certified site, with the goal of matching the thermo-mechanical load and the processing history. Once again modeling is the tool of choice, and a possible screw parameter for the GMP extruder has to be computed prior to the process transfer.

In summary, the proposed rational framework makes it possible to perform the product and process development for enabling formulations made *via* hot-melt extrusion within less than a year. Similar considerations can be applied to enabling formulation made *via* spray drying. This will be the focus of our future research.

## Abbreviations

API: Active pharmaceutical ingredient
ASD: Amorphous solid dispersion
BCS: Biopharmaceutical classification system
CFD: Computational fluid dynamics
CMA: Critical material attributes
CPP: Critical process parameters
CQA: Critical quality attribute
CQA: Critical quality attributes
DCS: Developability classification system
DDS: Drug delivery system
DEM: Discrete element method
DES: Dielectric spectroscopy
DMA: Dynamic mechanical analysis
DoE: Design of experiments
DSC: Differential scanning calorimetry
ESR: Electron spin resonance
FH: Flory-Huggins
FIH: First-in-human
GI: Gastro-intestinal
GMP: Good manufacturing practice
GPU: Graphical processing unit
HME: Hot melt extrusion
ICH: International council for harmonization of technical requirements for pharmaceuticals for human use
IR: Infrared
IVIVC: *In vitro*–*in vivo* correlations
MD: Molecular dynamics
NCE: New chemical entities
NMR: Nuclear magnetic resonance
PAT: Process analytical technology
PBPK: Physiologically based pharmacokinetics
PK: Pharmacokinetics
PSD: Particle size distribution
QTPP: Quality target product profile
RH: Relative humidity
RTD: Residence time distribution
SD: Spray drying
SMEC: Specific mechanical energy consumption
SPH: Smoothed particle hydrodynamics
TSE: Twin screw extruder
VCM: Vacuum compression molding
XPS: Extended particle system
XRPD: X-ray powder diffraction
